# Nicotinamide Riboside, an NAD + Precursor, Protects Against Cardiac Mitochondrial Dysfunction in Fetal Guinea Pigs Exposed to Gestational Hypoxia

**DOI:** 10.1007/s43032-023-01387-6

**Published:** 2023-11-13

**Authors:** Loren P. Thompson, Hong Song, Jamie Hartnett

**Affiliations:** Department of Obstetrics, Gynecology and Reproductive Sciences, University of Maryland, Baltimore, School of Medicine, 655 W. Baltimore St., Baltimore, MD 21201 USA

**Keywords:** Fetus, Heart, Placenta, Hypoxia, Mitochondria, Acetylation, Nicotinamide, Sirtuin, NAD

## Abstract

Gestational hypoxia inhibits mitochondrial function in the fetal heart and placenta contributing to fetal growth restriction and organ dysfunction. NAD + deficiency may contribute to a metabolic deficit by inhibiting oxidative phosphorylation and ATP synthesis. We tested the effects of nicotinamide riboside (NR), an NAD + precursor, as a treatment for reversing known mitochondrial dysfunction in hypoxic fetal hearts. Pregnant guinea pigs were housed in room air (normoxia) or placed in a hypoxic chamber (10.5%O_2_) for the last 14 days of gestation (term = 65 days) and administered either water or NR (1.6 mg/ml) in the drinking bottle. Fetuses were excised at term, and NAD + levels of maternal liver, placenta, and fetal heart ventricles were measured. Indices of mitochondrial function (complex IV activity, sirtuin 3 activity, protein acetylation) and ATP synthesis were measured in fetal heart ventricles of NR-treated/untreated normoxic and hypoxic animals. Hypoxia reduced fetal body weight in both sexes (*p* = 0.01), which was prevented by NR. Hypoxia had no effect on maternal liver NAD + levels but decreased (*p* = 0.04) placenta NAD + levels, the latter normalized with NR treatment. Hypoxia had no effect on fetal heart NAD + but decreased (*p* < 0.05) mitochondrial complex IV and sirtuin 3 activities, ATP content, and increased mitochondrial acetylation, which were all normalized with maternal NR. Hypoxia increased (*p* < 0.05) mitochondrial acetylation in female fetal hearts but had no effect on other mitochondrial indices. We conclude that maternal NR is an effective treatment for normalizing mitochondrial dysfunction and ATP synthesis in the hypoxic fetal heart.

## Introduction

Gestational hypoxia is a significant pregnancy complication that affects both the mother and fetus. Chronic intrauterine hypoxia inhibits mitochondrial function and generates oxidative stress in both the placenta and fetal organs including the heart [1–4]. Mitochondrial function is central to the generation of the cell’s energy supply, which is regulated by the delivery of NADH to complex I in the respiratory chain for the generation of ATP. Hypoxia inhibits mitochondrial respiration by reducing oxygen availability for ATP synthesis, increasing ROS (reactive oxygen species) formation, and reducing NAD + levels, all of which inhibit oxidative phosphorylation [5]. Thus, we hypothesize that gestational hypoxia reduces NAD + levels in fetal organs, which generates a significant metabolic deficit affecting fetal growth, development, and organ function.

NAD + (nicotinamide adenine dinucleotide) is the most abundant molecule in the body [6] and a cofactor important in hundreds of enzymatic reactions including oxidative phosphorylation (OXPHOS) [7]. It also activates NAD + -dependent enzymes such as mitochondrial sirtuins (SIRTs), for deacetylation of proteins of the respiratory chain [7, 8].

Several dietary NAD + substrates including nicotinamide (NAM), nicotinic acid, nicotinamide mononucleotide (NMN), and nicotinamide riboside (NR) can be delivered to the cell and converted to NAD + by ectoenzymes (CD38) at the plasma membrane and/or enter the cell via membrane transporters (e.g., ENTs and SLCs) and then converted to NAD + through its salvage pathway [6]. Intracellular NR generates NAD + after being converted to NMN by NRK1/2 (nicotinamide riboside kinase). NR is the preferred NAD + precursor, over other precursors, because of its high oral bioavailability, greater efficiency in cellular uptake, and increases NAD + levels in humans with fewer side effects, such as flushing [6, 8, 9].

Few studies have investigated the role of NAD + in pregnancy and fetal organ function and, particularly, under conditions of chronic hypoxia. Yet, NAD + deficiency plays a causal role in aging [10], obesity [11], and heart disorders [12, 13], generating multiple clinical trials (clinicaltrial.gov) on the treatment benefits of NAD + precursors. In the adult heart, NAD + deficiency is causally linked to mitochondrial and contractile dysfunction via hyperacetylation of mitochondrial proteins [13–17]. In early development, NAD + deficiency contributes to congenital malformations in several organs including the heart [18] and plays a role in preeclamptic pregnancy, where both maternal hypertension and fetal growth restriction are reversed with NR treatment in a mouse model of elevated soluble Flt1 levels [19].

Chronic intrauterine hypoxia impairs contractile [20, 21] and mitochondrial [22–25] function in fetal hearts, and downregulates fetal cardiac mitochondrial respiratory complex I, III, and V subunits, and alters complex I and IV activity rates [25]. Chronic hypoxia inhibits oxygenases of the NAD + synthetic tryptophan pathway [26], as well as, sirtuin activity of fetal cardiac mitochondrial proteins [27]. Sirtuin 3 (SIRT3) is an NAD + -dependent deacetylase [28] and the most important mitochondrial SIRT whose activity regulates protein acetylation of respiratory complex subunits I and IV [29]. SIRT3 knockout mice exhibit hyperacetylation of OXPHOS enzymes, reduced ATP, and cardiac hypertrophy [30]. Thus, the effects of hypoxia on NAD + levels identify an important link between mitochondrial respiration, SIRT3 activity, and NAD + regulation [7]. The aim of this study is to investigate the role of NR administration as a treatment for normalizing conditions of impaired mitochondrial respiratory function in the hypoxic fetal heart [22–25].

## Methods

### Animal Model

Hartley guinea pigs were purchased from Elm Hill Labs (Chelmsford, MA) and housed under conditions of 12 h of light/12 h of dark with free access to food and water. Timed-mated pregnant Hartley guinea pigs were exposed to either normoxia (room air, *N* = 27) for the entire pregnancy or placed in a hypoxic (10.5%O_2_, *N* = 30) environment for 14d duration from 50- to 64-day gestation (term = 65-day gestation). This gestational age of HPX exposure corresponds to the period of rapid fetal growth, post organogenesis, placental maturation, and greatest oxygen utilization by the guinea pig fetus [31]. The hypoxic chamber consists of a plexiglass box where animals are housed in individual containers with food and water. Oxygen levels in the chamber are reduced to 10.5%O_2_ with N_2_ gas mixed with room air, monitored with an O_2_ sensing probe (Reming Bioinstruments, Redfield, NY) and regulated by a servo-controlled feedback regulator. Excess CO_2_ and H_2_O vapor are scrubbed with soda lime and silica gel, respectively, placed in separate containers. All animals are housed in a temperature-controlled room with a light/dark cycle every 12 h. Normoxic and hypoxic pregnant animals were administered ad libitum either water or nicotinamide riboside (NR) in their drinking bottles for the last 14 days of pregnancy (50- to 64-day gestation). Food (g/day/kg) and water (ml/day/kg) intake rates were measured every other day. NR was administered at a single concentration of 1.6 mg/ml in the water bottle, and achieved a dose range of 117–151 mg/kg/day determined by the animal’s water intake rate. This dose was based on acquiring a significant increase in maternal liver NAD + levels, which confirmed oral bioavailability in pregnant sows. Dose ranges of 15 mg/kg/day in humans [9] and 185–300 mg/kg/day in rodents [8, 9] are considered safe and effective. Nicotinamide riboside chloride salt was provided as a gift from ChromaDex, Inc (Irvine, CA).

### Extraction of Fetal Tissue

At 64-day gestation, pregnant sows were anesthetized with ketamine (80 mg/kg, s.c.) and xylazine (6 mg/kg, s.c.), administered a lidocaine (2%) skin block, and an abdominal incision and hysterotomy performed for fetal extraction. Anesthetized fetuses were removed from the uterus; the placenta detached from uterine wall, fetuses, and placentas weighed. Fetal organs (heart, liver, kidney, and brain) of untreated and NR-treated normoxic and hypoxic animals were extracted and weighed and normalized to their respective fetal body weight as relative organ weight (organ wt/fetal body wt). Placentas and fetal heart left ventricles were excised and immediately frozen in liquid N_2_ and stored in − 80 °C until the time of the assay. The right lobe of the maternal liver was also excised and frozen for NAD + analysis.

### Mitochondrial Protein Extraction

To obtain mitochondrial fractions of fetal heart ventricles, 20–30 mg frozen pieces were ground with a mortar and pestle in liquid N_2_ and resuspended in 800 μl of ice-cold homogenization buffer. The mitochondrial fraction was isolated by a standard differential centrifugation method [22, 24]. The pellet containing the mitochondrial fraction was resuspended in 1 × RIPA Lysis Buffer (Millipore, Burlington, MA) and supplemented with 1 × protease inhibitor cocktail tablet (Roche Diagnostics, Munich, Germany). This procedure generates mitochondrial fractions [32] without contamination. Protein concentrations were determined by the Bio-Rad Protein Assay (Bio-Rad, Hercules, CA). Mitochondrial fractions were used for Western immunoblot and measurement of SIRT3 and CIV enzyme activities.

### NAD + Analysis

NAD + levels were measured using a NAD/NADH-Glo™ Assay (Promega, Madison, WI). Briefly, 10–20 mg frozen maternal liver, placenta, and fetal heart ventricles were ground into powder in liquid N_2_ and added to PBS/bicarbonate buffer according to the manufacturer’s instructions. An aliquot was obtained for determination of protein concentration using the Pierce 660 nm Protein Assay (ThermoFisher, Lanham, MD) method. NAD/NADH-Glo™ Detection Reagent was added to each microplate well, shaken, and incubated at room temperature for 30–60 min, and luminescence was recorded using a luminometer. Tissue NAD + content was measured as nMoles/mg protein (liver) or pMoles/mg protein (placenta and fetal heart) from the standard curve.

### Western Immunoblot

Isolated mitochondrial protein (20–30 µg) were loaded onto precast 4–12% Bis-Tris mini gels (Invitrogen, Waltham, MA) for gel electrophoresis and then transferred to a PVDF membrane. Acetylation of mitochondrial proteins was detected by anti-acetylated-lysine (Ac-K^2^-100) (1:1000, Cell Signaling Technology). For mitochondrial fractions of fetal heart ventricles, VDAC/Porin (1:1000)(Abcam, Cambridge MA) was used as the loading control [27]. Density values of each of the sample bands were normalized to their corresponding loading control as relative expression. A peroxidase-conjugated secondary antibody (SeraCare Life Sciences, Gaithersburg, MD) was used for all immunoblots.

### SIRT 3 Activity Assay

SIRT3 is a mitochondrial NAD + -dependent deacetylase [28]. SIRT3 activity of mitochondrial fractions of fetal heart ventricles was measured using the SIRT3 Activity Assay Kit (Abcam, Cambridge MA) per the manufacturer’s protocol. Briefly, 50 µg of mitochondrial protein was added into individual microplate wells containing a designated amount of fluorescence-labeled acetylated substrate peptide, nicotinamide adenine dinucleotide (NAD), and the developer. Fluorescence intensity was measured (Excitation at 350 nm/Emission at 450 nm) for 30 min at 1-min intervals by the microplate reader (BioTek, Winooski, VT). Enzyme activity rate was calculated as ΔOD/min.

### Cytochrome c Oxidase (Complex IV Subunit) Activity

Complex IV (cytochrome c oxidase) activity is responsible for reduction of O_2_ to H_2_O and is a measure of the oxidative capacity of the respiratory chain. Cytochrome c oxidase activity of fetal heart ventricles was measured colorimetrically following the oxidation of reduced cytochrome c (ferrocytochrome c). Mitochondrial protein (4 μg) was selected for an optimal reaction rate and added to a 96-well plate containing the assay buffer (10 mM Tri-HCl, pH 7.0, and 120 mM KCl) plus 0.04 mM reduced cytochrome c (Sigma-Aldrich, St. Louis, MO) [33]. The OD values generated by oxidation of the reduced cytochrome c were measured as a decrease in absorbance at 550 nm in a 96-well plate reader (BioTek, Winooski, VT) at 10-s intervals. The reaction rates of each sample were directly determined from a tangent drawn on the reaction curve at the 3-min time interval [34]. Cytochrome c oxidase activity is measured as units/mg protein derived from the following equation, ΔOD/time)/*ε* × protein (mg), where *ε* = 7.04 mM^−1^ cm^−1^.

### Fetal Heart ATP Content

Tissue ATP content was measured in frozen fetal heart ventricles of each treatment group. Tissue lysates were generated by homogenizing tissue, sitting on ice, in 100 μl ATP Assay buffer with a Dounce homogenizer. ATP content was obtained using a Fluorometric ATP Assay kit (Abcam, Cambridge MA) by following the manufacturer’s protocol. Briefly, 50 μl of Reaction Mix from the assay kit was mixed with 50 μl of lysates in a 96-well microplate. Fluorescence of signal was detected at excitation at 535 nm and emission at 587 nm on a microplate reader at 5-min interval for 30 min (BioTek, Winooski, VT). Sample ATP levels were measured in duplicate and averaged. ATP content of each sample was derived from the standard curve, normalized to their mg protein, and calculated as nM/mg protein.

### Statistical Analysis

Data are expressed as means ± SE. Each *N* value represents a single fetus. A total number of 18 normoxia (NMX), 18 hypoxia (HPX), 9 NMX + NR, and 12 HPX + NR pregnant sows were generated to obtain male and female fetuses from each group. *N* values varied for each group because multiple fetuses from the same litter were needed to complete all assays due to the limitation of fetal heart tissue availability. Fetal body weight characteristics were generated from all of the fetuses used in the study. Statistical analysis was performed using Systat software (San Jose, CA). Comparisons between the four treatment groups were made using one-way ANOVA. If differences between means were significant (*p* < 0.05), a post hoc analysis using the Holm-Sidak method was performed to identify differences between groups. For Western blot analysis, comparisons between two groups loaded onto a single gel were made using Student’s *t* test.

## Results

### Effects of Hypoxia and NR Treatment on Fetal Body and Organ Weights

Food intake rates were similar between all 4 treatment groups (Table 1). Water intake rates were similar in all but HPX + NR, which was significantly decreased (*p* = 0.01). The decrease in volume intake in HPX animals compared to the other 3 treatment groups (i.e., NMX, HPX, NMX + NR) accounted for the significant decrease in the NR dose in HPX animals.

**Table 1:** Effects of chronic hypoxia and nicotinamide riboside (NR) on fetal organ weights

	Control		Nicotinamide riboside	
Pregnant sows	NMX	HPX	NMX	HPX
*N* values	18	18	9	12
Food Int (g/d/kg)	41.7 ± 1.8	42.1 ± 2.9	42.2 ± 3.30	37.7 ± 1.7
Volume Int (ml/d/kg)	100.6 ± 12.3	131.9 ± 18.5	94.9 ± 11.0	76.6 ± 6.0*
NR dose (mg/d/kg)	0.0	0.0	151.0 ± 16.8	117.0 ± 7.6*
Fetal weights	Males			
	Control		Nicotinamide riboside	
	NMX	HPX	NMX	HPX
*N* values	23	18	10	16
*Absolute Wt*				
FBW(g)	92.7 ± 3.19	81.2 ± 1.63*	94.8 ± 4.94	89.0 ± 3.20
Plac Wt (g)	4.65 ± 0.23	4.92 ± 0.28	4.71 ± 0.25	5.61 ± 0.33
FHt (g)	0.53 ± 0.02	0.45 ± 0.01†	0.53 ± 0.03	0.51 ± 0.01
FBr (g)	2.63 ± 0.03	2.51 ± 0.04	2.66 ± 0.05	2.60 ± 0.04
*Relative Wt*				
Plac/FBW	0.0499 ± 0.0016	0.0607 ± 0.0035†	0.0498 ± 0.0017	0.0633 ± 0.0030†
FHt/FBW	0.0058 ± 0.0002	0.0055 ± 0.0002	0.0056 ± 0.0002	0.0058 ± 0.0002
FBr/FBW	0.0289 ± 0.0009	0.0310 ± 0.0005	0.0285 ± 0.0011	0.0295 ± 0.0008
	Females			
	Control		Nicotinamide riboside	
	NMX	HPX	NMX	HPX
*N* values	19	18	12	15
*Absolute Wt*				
FBW(g)	98.2 ± 4.04	79.1 ± 3.78*	88.6 ± 5.23	85.8 ± 4.31
Plac Wt (g)	4.96 ± 0.21	4.73 ± 0.21	4.48 ± 0.18	5.12 ± 0.20
FHt (g)	0.56 ± 0.02	0.47 ± 0.02†	0.50 ± 0.02†	0.47 ± 0.02†
FBr (g)	2.58 ± 0.03	2.43 ± 0.06	2.51 ± 0.05	2.56 ± 0.05
*Relative Wt*				
Plac/FBW	0.0507 ± 0.0012	0.0611 ± 0.0030†	0.0542 ± 0.0029	0.0613 ± 0.0032†
FHt/FBW	0.0057 ± 0.0001	0.0062 ± 0.0004	0.0060 ± 0.0003	0.0056 ± 0.0001
FBr/FBW	0.0268 ± 0.0009	0.0315 ± 0.0010†	0.0313 ± 0.0022	0.0306 ± 0.0012

*NMX* normoxia, *HPX* hypoxia, *F* fetal, *BW* body wt, *Ht* heart, *Br* brain, *Plac* placenta, *Int* intake

Comparisons between treatments using one-way ANOVA with statistical significance (*P* < 0.05) indicated by an asterisk from their respective NMX control. If *P* < 0.05, then a post hoc analysis using Holm-Sidak method was performed to identify differences between groups

*p < 0.05, †*p* < 0.01 vs NMX control

Chronic hypoxia generates a significant decrease (*p* = 0.01) in fetal body weight along with an increase (*p* < 0.01) in the relative placenta weight in both sexes (Table 1), demonstrating a compensatory response of the placenta to HPX. NR treatment normalized the decrease in body weight in HPX fetuses of both sexes compared to NMX but had no effect on body weight of NMX fetuses. In male fetuses, hypoxia also decreased (*p* = 0.005) absolute fetal heart, but had no effect on relative fetal organ weights compared to NMX controls. NR treatment prevented the decrease in heart weight in HPX fetuses and were similar to NMX controls. In females, HPX decreased (*p* = 0.01) absolute fetal heart weight and increased (*p* = 0.008) relative brain weight. NR treatment did not prevent the decrease in fetal heart weight in HPX fetuses but did normalize the increase in relative fetal brain weight. The increase in fetal brain weight relative to body weight indicates a brain sparing effect to hypoxia as a mechanism for improving organ survival.

### NAD + Levels

**Fig. 1:**
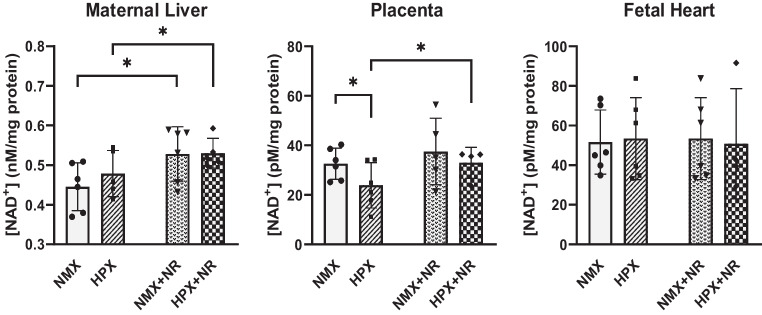
Tissue NAD + levels of maternal liver, placenta, and fetal heart ventricles in response to 4 treatments (normoxia (NMX), hypoxia (HPX, 10.5%O_2_, 14 days), NMX plus NR (nicotinamide riboside, 14 days) or HPX plus NR (14 days)). *p < 0.05. *n* = 6 in each group

Hypoxia had no effect on maternal liver NAD + levels compared to NMX controls (Fig. 1). However, oral administration of NR increased hepatic NAD + levels under conditions of both normoxia (*p* = 0.03) and hypoxia (*p* = 0.04). Hypoxia significantly (*p* = 0.04) reduced NAD + levels in placentas by 27% compared to NMX controls. NR treatment normalized NAD + levels of HPX placentas to levels similar to NMX controls but had no additional effect in NMX placentas. In contrast, HPX had no significant effect on NAD + levels in fetal heart ventricles. NR administration slightly elevated NAD + levels in HPX hearts although there was no significant difference from HPX untreated controls. The normalization of reduced NAD + levels in HPX placentas to normoxic levels indicates the bioavailability of oral maternal NR to the placenta.

### SIRT3 Activity Rate

**Fig. 2:**
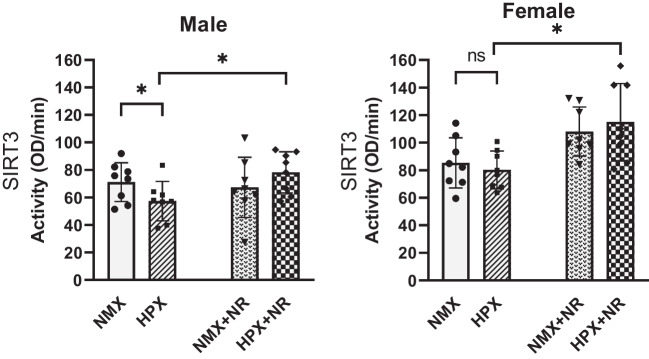
Effects of hypoxia and nicotinamide riboside (NR) on fetal heart SIRT3 activity. SIRT3 (sirtuin 3) activity rates of isolated mitochondria of male (left) and female (right) fetal heart ventricles. Activity rates were measured as OD/min. *p < 0.05. *n* = 8 in each group

To determine the effectiveness of NR administration on its NAD + -dependent molecular targets such as SIRT3, we measured the catalytic activity using isolated fetal heart mitochondria (Fig. 2). Chronic hypoxia decreased (*p* = 0.04) SIRT3 activity in males but not females compared to their NMX controls. NR administration increased (*p* = 0.006) SIRT3 activity in HPX males compared to the HPX untreated controls, despite a lack of change in NAD + levels. In females, hypoxia had no significant effect on SIRT3 activity although NR treatment increased (*p* = 0.004) SIRT3 activity in HPX hearts compared to untreated controls.

### Mitochondrial Acetylation Levels

**Fig. 3:**
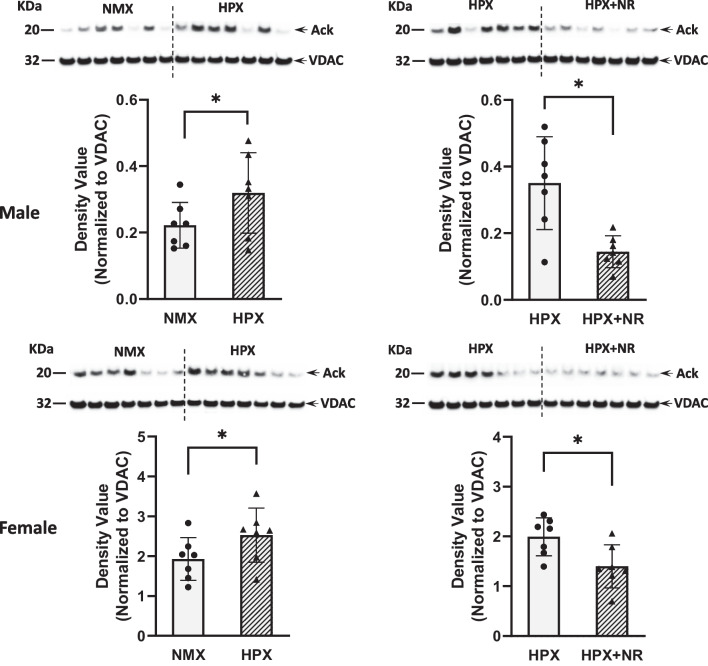
Effects of hypoxia and nicotinamide riboside (NR) on protein acetylation of isolated mitochondria from fetal heart ventricles from male (top) and female (bottom) animals exposed to NMX (normoxia), HPX (hypoxia, 10.5%O2, 14 days) or HPX plus NR (14 days). Western immunoblots (top) identify acetylated mitochondrial protein (Ack) at ~ 20 kDa. Each lane represents a different fetal sample. Graphic analysis below each blot illustrates the expression levels (density value) of acetylated protein for each treatment group relative to its own loading control (VDAC/Porin). No other proteins on the gel were acetylated to levels that were quantifiable. *p < 0.05. *n* = 7 in each group

Acetylation of proteins is a posttranslational modification that inhibits the activity of enzymes and targets mitochondrial complex subunits in the respiratory chain [35]. Protein acetylation was identified as strong bands of acetylated-lysine at ~ 20 kDa, which is associated with respiratory complex I (NDUFB8) in guinea pig fetal heart ventricle [27]. Hypoxia significantly increased acetylation in both male (*p* = 0.04) and female (*p* = 0.46) heart mitochondria (Fig. 3). Maternal treatment of NR reduced mitochondrial acetylation in both male (*p* = 0.002) and female (*p* = 0.01) HPX hearts compared to their untreated HPX controls. Maternal NR significantly (*p* = 0.005) reduced mitochondrial acetylation levels in NMX hearts of females but not males (data not shown).

### Cytochrome c Oxidase Activity (CIV) Rate

**Fig. 4:**
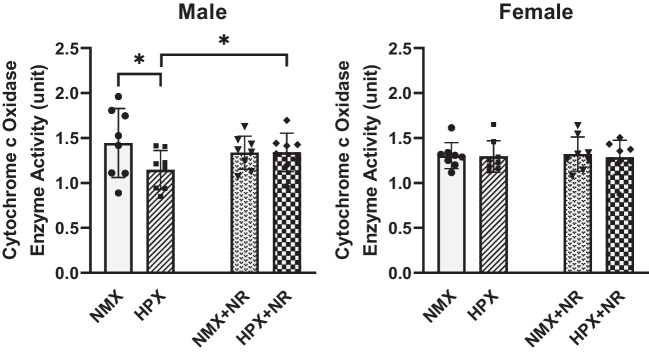
Effects of hypoxia and nicotinamide riboside (NR) on cytochrome c oxidase (Complex IV) activity rate of heart ventricles from male and female fetuses. Complex IV enzyme activity rates were compared between normoxia (NMX), hypoxia (HPX, 10.5%O_2_, 14 days), NMX plus NR (nicotinamide riboside, 14 days), and HPX plus NR (14 days). *p < 0.05. *n* = 8 in each group

Cytochrome c oxidase is a catalytic subunit of mitochondrial CIV. Hypoxia reduced (*p* = 0.037) CIV activity in male but not female hearts (Fig. 4). NR administration normalized the decrease in CIV activity in male fetal hearts to levels similar to normoxic levels. NR had no effect on CIV activity in female hearts.

### ATP Content

**Fig. 5:**
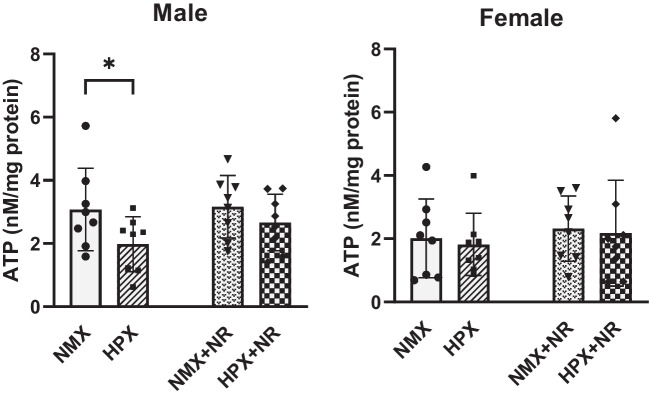
Effects of hypoxia and nicotinamide riboside (NR) on ATP content of heart ventricles from male and female fetuses. Tissue ATP content (nM/mg or pM/mg protein) was compared between normoxia (NMX), hypoxia (HPX, 10.5%O_2_, 14 days), NMX plus NR (nicotinamide riboside, 14 days), and HPX plus NR (14 days). *p < 0.05. *n* = 8 in each group

Chronic hypoxia significantly reduced (*p* = 0.034) ATP content in male but not female heart ventricles (Fig. 5). In HPX male hearts treated with NR, ATP levels were increased but did not reach significance (*p* = 0.07) compared to its untreated HPX controls. Hypoxia had no effect on ATP levels in female hearts and were unaltered by treatment with NR.

## Discussion

This study demonstrates that oral administration of NR to the pregnant sow normalizes placental NAD + levels and fetal heart mitochondrial respiratory protein dysfunction in response to hypoxia. This identifies a novel treatment of protection against gestational hypoxia in both the placenta and fetal heart. The protective effects of NR in hypoxic fetal hearts are hypothesized to be mediated by activation of mitochondrial SIRT3 and hypoacetylation of mitochondrial proteins.

Exposure to hypoxia in late gestation reduces fetal growth despite a compensatory increase in relative placental weight, suggestive of placental insufficiency. Fetal growth restriction in hypoxic fetuses is likely attributed to the effects of hypoxia on placental function and/or fetal growth mechanisms rather than nutrient deficiency since food intake rate was unaffected by hypoxia exposure. In the current study, the normalization of fetal body weight in hypoxic male fetuses by NR could be mediated by improving placental function via its increase in placental NAD + level and improving cell metabolism [36], mitochondrial respiratory function [24], and trophoblast survival [37–40]. In adverse pregnancies, NAD + deficiency reduces SIRT activity and initiates decreased placental function contributing to increased oxidative stress, inflammation, altered placental metabolism, and decreased trophoblast survival [36]. Chronic intrauterine hypoxia reduces mitochondrial respiratory complex I and IV activities in the placenta [24] and inhibits endovascular invasion of trophoblast cells [37–40] concomitant with fetal growth restriction. Restoring NAD + levels in hypoxic placentas improves trophoblast metabolism and potentially nutrient transport functions because of NAD + ’s ubiquitous roles in metabolic reactions [41]. Further, in preeclampsia mouse models, nicotinamide (NAM, an NAD + precursor) administration normalized NAD + deficiency and prevented embryo growth restriction via placental mechanisms associated with inhibiting endothelin-1 receptor activation, improving endothelial function and perfusion [19, 42].

NR may also have a direct effect on the fetus and its growth since NR increased ATP synthesis and normalized mitochondrial SIRT3 and CIV activities in hypoxic fetal hearts. Since maternal NR targets the fetal heart, other fetal organs such as skeletal muscle [43] could also be affected by NR through NAD + ’s action on glycolysis and TCA cycling [18, 19, 42, 44]. The ability of NR to alter mitochondrial respiratory mechanisms in the fetal heart is likely to impact metabolic processes other fetal organs in contributing to normalizing fetal body weight.

There was a differential response of NR on NAD + levels in the maternal liver, placenta, and fetal heart. The increase in hepatic NAD + levels with NR demonstrates the bioavailability of oral NR [reviewed in 8] in the pregnant sow. However, in fetal heart, NAD + levels were unchanged by hypoxia alone or altered with NR administration despite having effects on both cardiac enzymatic activities and ATP levels. The ability of the fetal heart to maintain a stable NAD + pool through its salvage pathways may occur via degradation of NAM, converted to NMN by NAMPT (NAM phosphoribosyltransferase) and then back into NAD + [45–47]. The heart has the highest level of NAD + content compared to other tissues such as liver, kidney, and skeletal muscle [48], necessary for supporting its high oxidative requirements [49, 50]. Seventy percent of the cardiac NAD + pool is contained within the mitochondria [47, 49, 50] compared to neurons and hepatocytes [51]. Further, heart NAD + levels remain stable even in the presence of reduced cytoplasmic NAD + [49] due to a difference in compartmentation between the mitochondria and cytosol [11, 52]. Thus, in the current study, any small changes in cardiac NAD + content generated by hypoxia and/or maternal NR may be unable to be detected because of the heart’s high capacity to maintain a stable pool. Further, both heart [9] and brain [16] nicotinic acid adenine dinucleotide (NAAD) [9], a major metabolite of NAD + , has been shown to increase in the absence of changes in NAD + , identifying a more sensitive index for assessing changes in NAD + levels. Yet, NR increased fetal cardiac NAD + -dependent SIRT3 activity, CIV activity, and ATP levels in males and SIRT3 activity in females despite the lack of measurable changes in NAD + levels in hypoxic or NR-treated fetal hearts.

The cardioprotective role of SIRT3 activity is well established in adult heart disease [46, 53]. SIRT3 is the most important NAD + -dependent enzyme in normal heart function because of its role in mitochondrial acetylation of OXPHOS proteins [6] and stimulating TCA and β-oxidation [52]. In hypoxic fetal hearts, the decrease in SIRT3 activity impairs normal metabolic function as evidenced by its hyperacetylation of mitochondrial proteins associated with the respiratory chain [27]. CIV activity was also reduced by hypoxia in male hearts and normalized with NR treatment, which affects the electron flux along the respiratory chain. It is reasonable to suggest that maternal NR restores ATP levels in hypoxic fetal hearts by maintaining OXPHOS via stimulating SIRT3 activity, reversing hyperacetylation of mitochondrial proteins, and restoring CIV activity.

While contractile function of the fetal heart was not assessed, ATP levels were measured as a functional index of cardiac metabolism, which were decreased with hypoxia and normalized by NR treatment. In ischemic hearts, reduced ATP levels are mediated by reduced NAD + levels [54–57]. Hyperacetylation of mitochondrial proteins is considered an inducer of cardiac dysfunction [15–17]. In the fetal heart, chronic hypoxia increases the susceptibility to ischemia/reperfusion injury [58] and decreases ventricular function in fetal chickens [59]. Despite a compensatory increase in diastolic relaxation in hypoxic fetal guinea pig hearts [60], prenatally hypoxic offspring exhibit both reduced cardiac mitochondrial and contractile function [23], suggesting that fetal hypoxia alters underlying mitochondrial respiratory mechanisms contributing to functional deficits postnatally. If hypoxia-induced hyperacetylation of respiratory proteins is limiting ATP synthesis, maternal NR may be cardioprotective against fetal hypoxia by maintaining a normal energy supply. In adult hearts, the effect of NR in ameliorating adverse cardiac remodeling and dysfunction is mediated by restoring NAD + levels [13]. The decrease in ATP levels in the hypoxic fetal heart identifies a metabolic deficit that could lead to decreased myocardial performance. Interestingly, female hearts were less affected by hypoxia with regards to ATP levels, and CIV and SIRT3 activity rates, perhaps identifying a favorable adaptive response to chronic hypoxia with regards to mitochondrial function [23, 25, 27].

**Fig. 6:**
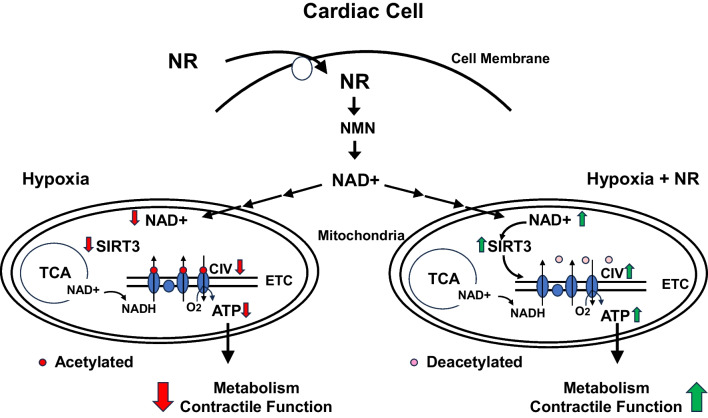
Schematic diagram of effect of nicotinamide riboside (NR) on mitochondrial function in fetal heart cell. NR enters the cell and is converted into NAD + via the salvage pathway. Hypoxia can decrease NAD + levels by inhibiting oxygenases of the tryptophan pathway [26]. Reduced mitochondrial NAD + levels decrease mitochondrial SIRT3 activity, which hyperacetylates mitochondrial respiratory proteins, decreases CIV activity and ATP levels. NR may reverse the effects of hypoxia on mitochondrial proteins by elevating mitochondrial NAD + levels, activating NAD + -dependent SIRT3, deacetylating mitochondrial respiratory proteins and normalizing CIV activity and ATP levels. Restoration of ATP levels by NR may sustain cardiac metabolism and function as a mechanism against fetal hypoxia. NAD + enters the mitochondria by being converted to NMN in the cytoplasm and transported across mitochondrial membranes and converted to NAD + . (SIRT3, mitochondrial sirtuin activity rate; CIV, complex IV activity rate; NMN, nicotinamide mononucleotide; NAD + , nicotinamide adenine dinucleotide; red arrows = directional change in response to hypoxia alone, green arrows = directional changes with NR treatment during hypoxia)

In conclusion, maternal NR administration provides protection against gestational hypoxia by mechanisms restoring the NAD + pool in the placenta and activating SIRT3 in the fetal heart. Figure 6 illustrates how maternal NR administration could normalize mitochondrial function in hypoxic mitochondria by stimulating SIRT3, deacetylating respiratory complexes, thereby increasing CIV activity and ATP levels. Given the effects of chronic hypoxia on the regulation of mitochondrial respiratory proteins in fetal hearts [13, 24, 25, 27], maternal treatment of NR may protect against programming effects of mitochondrial dysfunction in the prenatally-hypoxic offspring, which exhibits both decreased cardiac contractile and mitochondrial function [23].

## Data Availability

All data generated is included in this publication.
